# OnetoMap Meta-Data: Healthcare Analytics Through Research

**DOI:** 10.7759/cureus.66763

**Published:** 2024-08-13

**Authors:** Nadayca Mateussi, Haroon Janjua, Emily A Grimsley, Melissa Kendall, Tyler Zander, Ricardo Pietrobon, Paul C Kuo

**Affiliations:** 1 SporeData, Durham, USA; 2 Surgery, University of South Florida Morsani College of Medicine, Tampa, USA

**Keywords:** clinical research databases, machine learning, analytics, meta-data, data

## Abstract

Introduction: Big Data has revolutionized healthcare research through the three Vs: volume, veracity, and variety. This study introduces the OnetoMap meta-data repository, a centralized inventory developed in collaboration with the University of South Florida's Department of Surgery.

Methods: The repository offers extensive details about each database, including its primary purpose, available variables, and examples of high-impact research utilizing these databases. It aims to create a centralized inventory, enabling researchers to locate and link relevant datasets efficiently. Each dataset is described using standardized criteria to ensure clarity and usability, such as data type, source, collection methods, and potential linkages to other datasets.

Results: Currently, the OnetoMap repository contains descriptions of 49 datasets, with ongoing updates to include new datasets and additional data years. These datasets include a range of data types, including cross-sectional and longitudinal, gathered through claims, registries, electronic health records, and surveys. The repository is hosted on GitHub, enabling version control, collaboration, and open access. Effective search functionalities and descriptive categorization enhance the findability of datasets.

Discussion: The data repository includes comprehensive records of patient health statuses, socioeconomic profiles, hospital structures, and physician practices, enabling nuanced interventions and addressing complex healthcare needs. It also promotes interdisciplinary research and accelerates novel discoveries by providing a centralized source of diverse data and facilitating collaboration among research teams.

Conclusion: The OnetoMap meta-data repository represents a significant advancement in healthcare research by providing a centralized, detailed, and easily accessible repository of clinical research databases. Future directions include implementing automatic annual updates of datasets, exploring automatic dataset linkage, providing monthly updates on published research, creating a user chat space for enhanced collaboration, and developing code applets for simplified data analysis. These efforts will ensure that the repository remains current, functional, and accessible, ultimately facilitating new discoveries and insights in healthcare outcomes research.

## Introduction

Big Data has significantly augmented research endeavors across various fields, with healthcare research benefiting greatly. By leveraging the power of Big Data and predictive analytics, researchers can perform sophisticated analyses and generate actionable insights. The three Vs of Big Data (volume, veracity, and variety) have transformed healthcare research to a new level [1]. Massive amounts of data are generated at high frequency, containing an array of attributes. It is no longer surprising that sophisticated data science algorithms can be implemented on open-source platforms, with many free training resources available to the research community [2].

All research projects begin with a research question, which lays the groundwork to develop and implement a detailed and conclusive analysis. In the conception phase, the viability of the research project is determined, and the dataset is chosen. This step also gives us the ability to realize study limitations, which are generally due to the availability of data in terms of timeline, predictors, geography, and other reasons. The conception phase has many implications for the project’s final result, including but not limited to the organization and selection of the suitable dataset, developing a data analysis strategy that encompasses data cleaning, preprocessing, modeling, and ultimately presenting the research findings persuasively [3]. This study aims to introduce the OnetoMap meta-data repository, a centralized inventory developed to enhance healthcare research by providing detailed descriptions of various datasets, enabling efficient dataset linkage, and promoting collaborative research.

## Materials and methods

We created a GitHub wiki to describe the databases in order to make the information available to the public. To create the GitHub page and GitHub wiki, we followed the several straightforward steps described in the GitHub documentation [4,5].

Structure of dataset description

**Figure 1:**
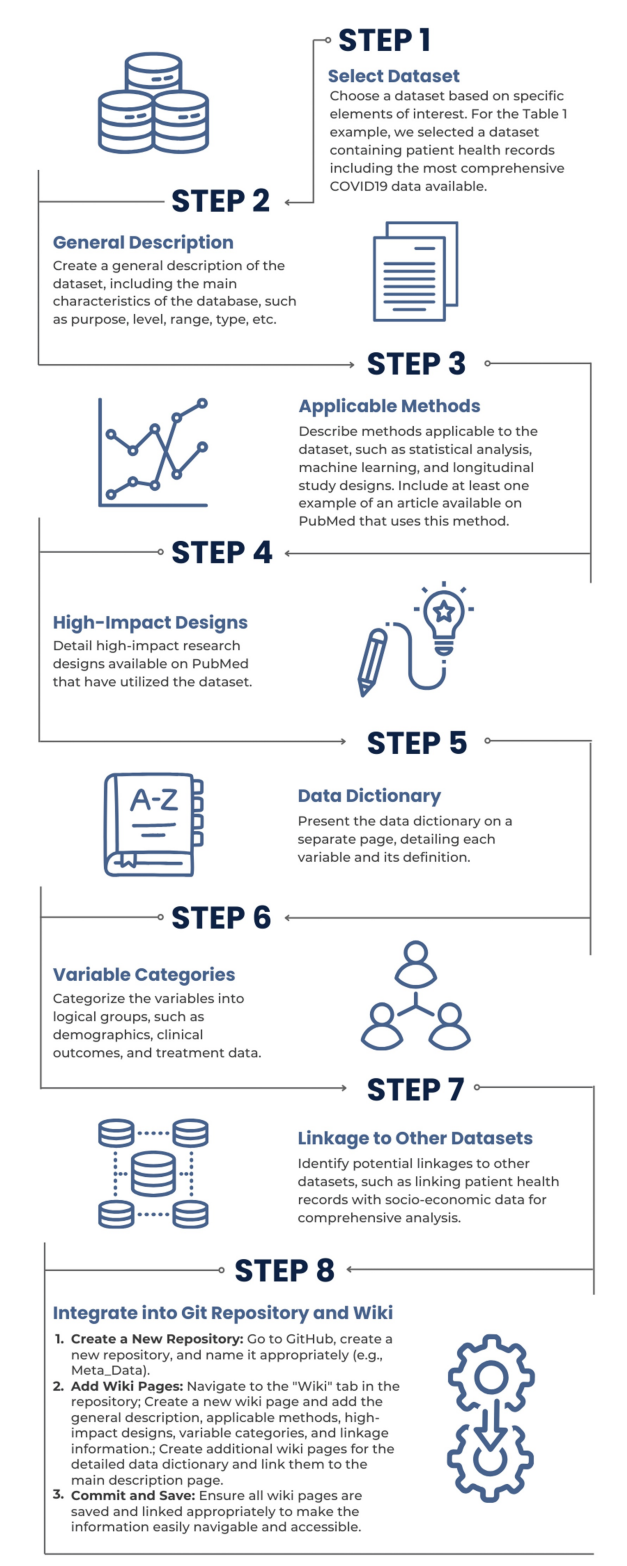
A step-by-step example showing how a specific dataset is described and integrated into the Git repository

To create the OnetoMap Git repository and insert dataset details, we began by selecting datasets of interest based on specific elements, such as the type of data available. We provided an overview of each dataset using information from data documentation, dictionaries, public use files (PUFs), and PubMed entries. We described all datasets following a set of criteria aiming to ensure that researchers have a good overview of the dataset of interest (Figure 1). We defined these criteria based on key elements such as data collection, years available, and variable type. Overall, we used the following fields in the description of the datasets: (1) general description (including data type, source, minimal level of collection of data, and geographic location of the data collection sites), (2) applicable methods, (3) high-impact designs, (4) data dictionary (which we present in details as a separate page), (5) variable categories, and (6) linkage to other datasets. A detailed explanation of each field is available in Table 1.

**Table 1:** Description of the description fields for each dataset, including a database as an example OMOP-CDM: Observational Medical Outcomes Partnership-Common Data Model

Field	Description	Example based on N3C database
1. General description		
a. Database primary purpose	The primary purpose of the database	Offer a comprehensive and centralized data resource to enable research teams to study COVID-19 and its associated properties
b. Overall data type	Type of document where the data is presented (e.g., hospital expenditures, demographics)	Health outcomes
c. Dataset type	Type of register of the dataset considering the focused subject and the period (i.e., longitudinal, cross-sectional)	Longitudinal
d. Data source	Type of source from where the data was extracted (e.g., clinical trials, claims)	Electronic health records (EHR)
e. Data level	Level of collection of data	Patient level
f. Geographic location of the data collection sites	Country and institutions from where data was collected	Currently, 98 institutions [6] have executed a data transfer agreement (DTA) with the National Center for Advancing Translational Sciences (NCATS)
g. Sponsor, manager, or home institution	Institutions involved in the funding, management, and maintenance of the project	National Center for Advancing Translational Sciences (NCATS)
h. Date range	Time interval of data register and/or availability	From January 1, 2018, to the latest data partner extraction date
i. Geolocation data	Geographical location information available (e.g., zip codes, ZCTA, county)	Patient zip codes accessible within the limited dataset
j. Dates	Dates availability (day, month, year)	Available under the limited dataset
k. Hospital identifiers	Any information that identifies the hospitals involved in the dataset	Synthetic data partner ID
l. National provider identifier (NPI)	If available	No
m. Physician identifiers	Any information that identifies the physician involved in the database	Synthetic provider ID
n. Longitudinal tracking	The method employed by the dataset to monitor patients both within and across hospitals, as well as to track providers	Track patients both within and across participating hospitals at office, inpatient, outpatient, and emergency department levels. Additionally, track providers across the hospitals that currently provide the provider ID, occurrences (e.g., visit, procedure), and the IDs mentioned above
o. Financial variables	Financial-related information available in the dataset	None
p. Clinical areas of interest	OMOP-CDM concepts that classify the database	All clinical areas
q. Number of records	The total count of individual entries or data points contained within a dataset	By June 2024, the N3C held information on 22.7 million anonymized patients, with 1.8 billion visits, more than 8.8 million COVID-positive cases, 3.3 billion clinical observations, 16.1 billion lab results, 5.1 billion medication records, and 1.3 billion procedures
r. Variables that are uniquely present in this dataset	Unusual type or exclusive information provided by the dataset	COVID-related variables, inpatient medications, general drug information, laboratory results, patient zip codes and dates in the limited dataset, and the linkage between inpatient, outpatient, emergency department, and office data
s. Database caveats and limitations	Limitations presented by the database	Hospitals cannot be identified per the Data Use Agreement (DUA); the dataset is restricted to patients who have undergone a COVID test; instances of a condition or procedure may be mapped to different OMOP-CDM concepts; and lab results values may not be consistent across hospitals
t. Other	Additional key information about the database	Depending on user and access requirements, three types of datasets are available, differing in content: Limited-Patient data includes PHI such as dates and zip codes; De-identified-PHI is altered to protect patient privacy; Synthetic-Data generated from the limited dataset that statistically resembles patient information but does not represent real patient data
2. Applicable methods	Examples of data science methods applicable to the dataset, as demonstrated in published articles	Regression models, propensity scores, sensitivity analysis, and machine learning
3. High impact designs	Examples of high-impact articles that have utilized the dataset	Evaluate COVID-19 severity and risk factors [7] and the use of different drugs [8]
4. Data dictionary	Detailed description of the content of the dataset's domains	The N3C data dictionary is available in the OnetoMap repository [9]
5. Variable categories	Set of variables included in the dataset	COVID-19 test results, patient demographics, death, visits, procedures, drug and device exposure, condition occurrence, measurements, and observations
6. Linkage to other datasets	Recommendations for linking datasets based on their attributes	Linkages can be made for any dataset that might have zip code information

## Results

**Figure 2:**
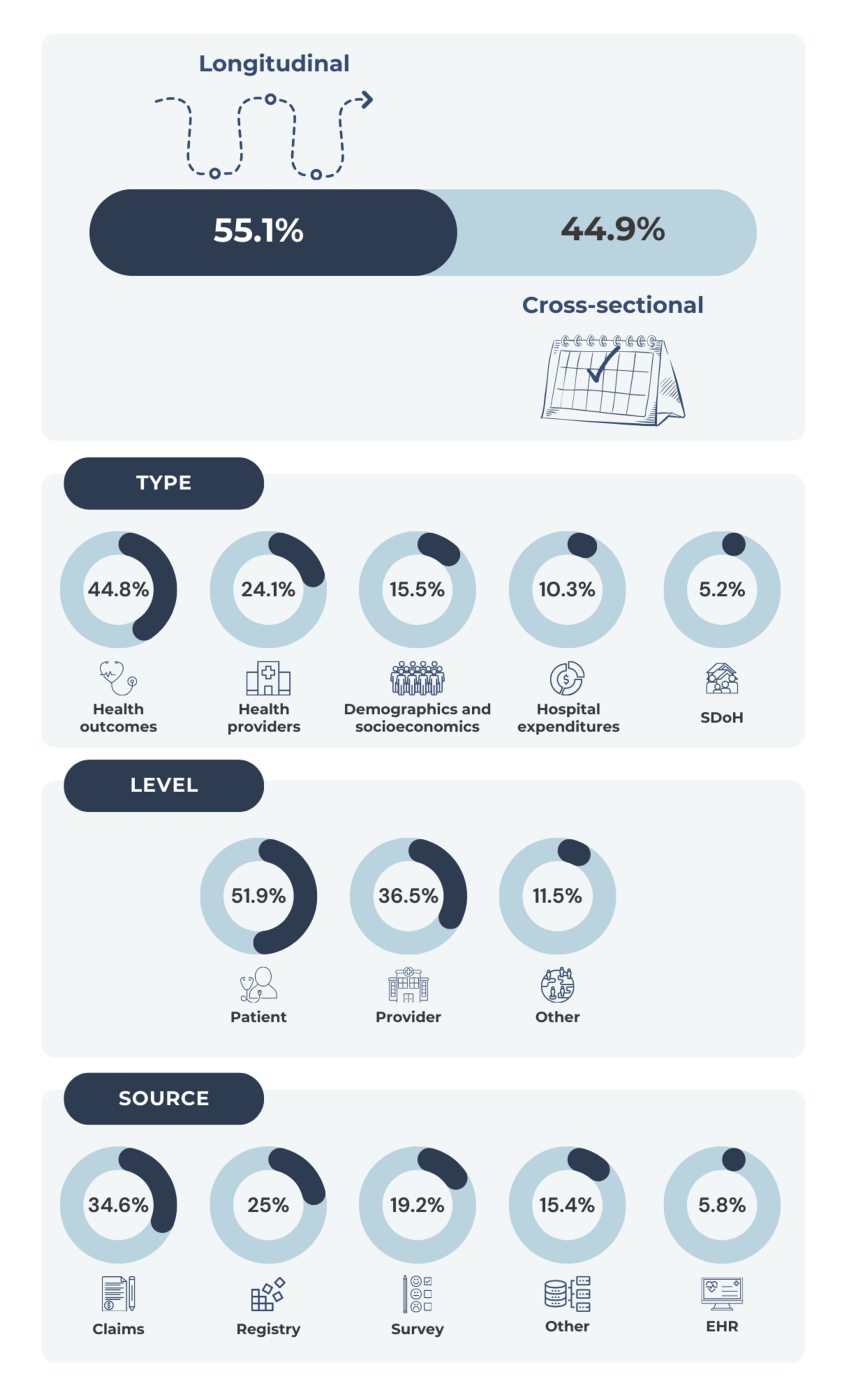
General characteristics and summary of the OnetoMap meta-data repository contents

Currently, 49 datasets are described in the OnetoMap™ meta-data repository, owned by OnetoMap LLC, which is constantly updated with new datasets and additional years of data (Table 2) [10]. Included datasets encompass a wide variety of data types, including longitudinal and cross-sectional datasets, gathered from claims, surveys, and electronic health records (EHR), encompassing patient health and socioeconomic demographics, hospital profiles, and physician details (Figure 2).

**Table 2:** List of the datasets currently stored in the OnetoMap repository

Datasets
AHA: American Hospital Association Annual Survey Database
AMGA: Medical Group Compensation and Productivity
Area Deprivation Index (ADI) Neighborhood Atlas
AHRF: Area Health Resources File
BCSC Hormone Therapy and Breast Cancer Incidence Dataset
BCSC Risk Estimation dataset
BCSC Risk Factors Dataset-Breast Cancer Surveillance Consortium
CBECS: Commercial Buildings Energy Consumption Survey
CDC SVI: Social Vulnerability Index
CHRR: County Health Rankings and Roadmaps
CMS HCRIS Hospital Cost Report
CMS Hospital Compare
CMS Open Payments
CMS Physician Compare
CMS Provider Utilization and Payment Data-Physician and Other Supplier Public Use Files
Dartmouth Atlas Project Data
EIG DCI: Economic Innovation Group Distressed Communities Index Data
Feeding America Datasets
FL AHCA: Florida Agency for Healthcare Administration Database
Gun Violence Archive
HCUP KID: Healthcare Cost and Utilization Project, Kids Inpatient Database
HCUP NIS: Healthcare Cost and Utilization Project, National (Nationwide) Inpatient Sample
HCUP NRD: Healthcare Cost and Utilization Project, Nationwide Readmissions Database
HCUP SASD: Healthcare Cost and Utilization Project, State Ambulatory Surgery Database
HCUP SEDD: Healthcare Cost and Utilization Project, State Emergency Department Databases
HCUP SID: Healthcare Cost and Utilization Project, State Inpatient Database
HIMSS IT Data: Healthcare Information and Management Systems Society
HSAF: Hospital Service Area Files
KHN: Kaiser Health News Data
Lown Institute Hospitals Index
MBSAQIP: Metabolic and Bariatric Surgery Accreditation and Quality Improvement Program
MEPS: Medical Expenditure Panel Survey
MIMIC-IV: Medical Information Mart for Intensive Care
N3C: National COVID Cohort Collaborative
NCDB: National Cancer Database Participant User Files
NHATS: National Health and Aging Trends Study
NORC: The Nonpartisan and Objective Research Organization NORC at the University of Chicago
NPDB: National Practitioner Data Bank Public Use Data File
NTDB: National Trauma Data Bank (TQP: Trauma Quality Program)
NSQIP: National Surgical Quality Improvement Program
NY SPARCS: Statewide Planning and Research Cooperative System
RAND-Hospital Data
Scottish Health and Social Care Open Data
SEER: Surveillance, Epidemiology, and End Results Program
STAR: Standard Transplant Analysis and Research Files
STS Intermacs de-identified datasets
Texas Hospital Discharge Data
Vermont Uniform Hospital Discharge Data
WRDS-Corporate Bond Database

Search procedure

The OnetoMap meta-data repository is located on GitHub [11], a web-based platform that provides hosting for software development projects using the Git version control system. GitHub is a powerful tool for developers and researchers alike. It provides access to a wealth of information and resources, including code, commits, issues, discussions, packages, and wikis. However, finding the information you need can be a challenge, especially when the repository or organization is large. Therefore, it is fundamental to know how to search effectively on GitHub.

To refine search results, GitHub allows the use of Boolean terms like “OR,” “AND,” and “NOT.” For example, "expenditures OR demographics OR EHR" will search for any of these concepts, while "claims AND hospital level" will find claims databases containing hospital-level data. The term "NOT" can be used to exclude specific keywords from the search, such as "hospital-level NOT claims," which will locate hospital-level data in all sources except claims. A detailed step-by-step search is available on the README page of the OnetoMap meta-data repository [12].

Findability

In the meta-data repository context, findability refers to the ease with which users can locate information or content on the GitHub repository. The findability of the OnetoMap meta-data repository is constantly being improved, aiming to ensure that users can quickly and easily find the data they are looking for without having to spend excessive time searching or navigating. This encompasses various factors, such as the organization and structure of the repository content, the use of search functionality, the labeling and categorization of content, and the use of descriptive and concise headings and titles.

License

All information in the OnetoMap meta-data repository (e.g., datasets description and dictionaries) is shared under the Creative Commons Attribution-NonCommercial-ShareAlike 4.0 International Public License [13]. However, each dataset listed in the repository retains its own original documentation, license, and Data Use Agreement, meaning they are not openly available. Parties interested in a research collaboration with the OnetoMap group can get in touch through the form available on the OnetoMap meta-data repository README page [12].

Data extraction, processing, and storage

**Figure 3:**
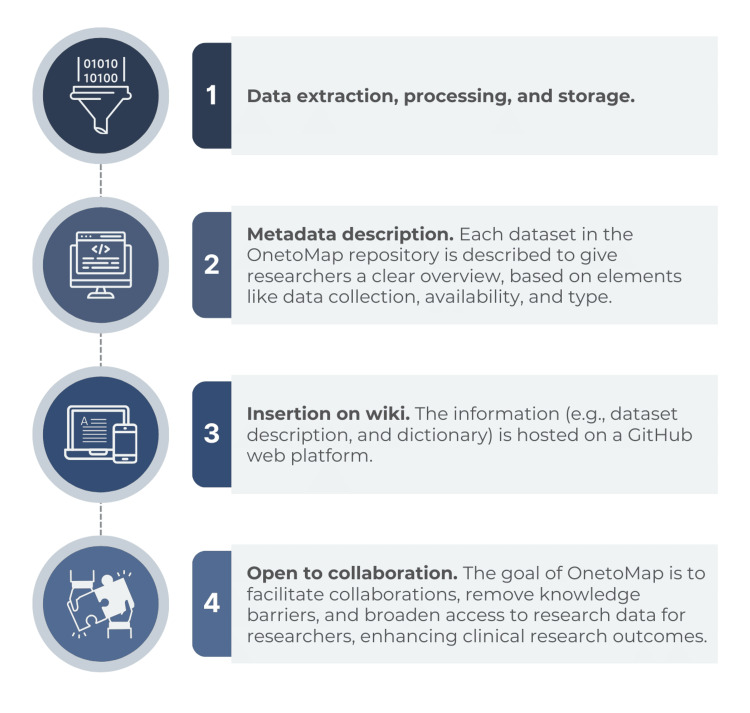
Diagram showing the workflow from data extraction to potential collaboration with other institutions

Once data is properly stored, we used the GitHub web platform to host the OnetoMap information (i.e., dataset description and dictionary) (Figure 3). Among the advantages of using GitHub to keep this information is (1) the possibility of version control, GitHub allows users to keep track of changes made to their repositories, making it easy to roll back to previous versions if necessary; (2) its collaborative characteristic, which allows the construction of a synergic network among users to improve the project collectively; and (3) the open-source nature of the repository, meaning information is freely available for anyone to access and potentially contribute.

## Discussion

OnetoMap Analytics strives to elucidate the complexities of healthcare delivery by employing Big Data analytics and advanced machine learning algorithms, thereby providing stakeholders with actionable insights to enhance the quality and efficiency of patient care. The extensive data repository detailed herein encompasses comprehensive records of patient health statuses, socioeconomic demographic profiles, hospital structures, and physician practices. This holistic perspective facilitates the development of nuanced and impactful interventions, effectively addressing the multifaceted needs of healthcare systems and communities.

Strengths

The OnetoMap repository boasts several significant strengths, as discussed in more detail below. Overall, it provides comprehensive dataset descriptions, including detailed data dictionaries and variable categorizations, ensuring researchers have a clear understanding of the available data. Also, its robust linkage capabilities allow researchers to connect various datasets, enhancing the depth and breadth of their analyses. In addition, OnetoMap promotes interdisciplinary research and collaboration, evidenced by the publications resulting from partnerships with the Department of Surgery at the University of South Florida. And finally, OnetoMap facilitates easy data access while ensuring ethical compliance.

Enhancement of collaborative research efforts

Clinical data repositories have demonstrated how centralized data resources can support multi-institutional data sharing and high-performance computing, critical for large-scale collaborative research projects [14]. In this context, by embracing a collaborative ethos, the OnetoMap meta-data repository facilitates collaborations among different research teams. Providing a centralized, comprehensive source of diverse healthcare data enables researchers from various institutions and disciplines to access and analyze shared datasets. This collaborative access promotes interdisciplinary research, accelerates the discovery of novel insights, and fosters the development of innovative solutions to complex healthcare challenges. The repository's shared resources and data standardization also ensure consistency in research methodologies and findings, enhancing the overall impact and reliability of collaborative research efforts [15-17]. Since its inception at Loyola and the University of South Florida, OnetoMap has significantly promoted interdisciplinary research and collaboration, facilitating 112 publications in collaboration with the Department of Surgery [18].

Improvement in data linkage and integration

Of the databases available in the repo, 67% can be linked to another dataset using different sets of variables, such as geolocation and identifiers, depending on the characteristics of the datasets of interest. The integration of distinct datasets offers significant benefits in terms of research comprehensiveness since it allows researchers to analyze multiple aspects of health, socioeconomic status, and other factors simultaneously, providing a more comprehensive understanding of patient populations and healthcare systems, as well as combining data from various sources (e.g., EHR, surveys, genomic data), enabling researchers to draw connections across different domains, enhancing the depth and breadth of their analyses [19,20]. In addition, the dataset integration may improve longitudinal studies by enabling the continuous tracking of individuals across different healthcare settings and over extended periods, which is a crucial capability for studying disease progression, treatment outcomes, and long-term health trends. By integrating data longitudinally, researchers can also identify patterns and trends that emerge over time, facilitating more accurate and dynamic models of health and disease [21]. Furthermore, the linkage of databases uncovers new insights through data merging that isolated datasets cannot provide. Merging data from multiple sources increases the statistical power of analyses, allowing for the detection of subtle effects and interactions that might be missed in isolated datasets [22]​. Moreover, integrated datasets can reveal new correlations and causal relationships that are not apparent when data is isolated [23]. Overall, the integration of diverse datasets not only enhances the comprehensiveness of research but also unlocks the potential for more detailed and longitudinal analyses, leading to novel insights and improved healthcare strategies.

Streamlining the ethical review process

Once the DUA is already established between individual datasets and the OnetoMap Analytics, the repository streamlines the ethical review process, leading to reductions in time and/or administrative burden for obtaining ethical approvals. Nevertheless, while this process accelerates research timelines, it still ensures compliance with ethical standards through a careful review process of the projects to be carried out prior to the execution of partnerships. 

It is important to note that while dataset descriptions and associated dictionaries are freely accessible, each dataset within the repository maintains its original documentation, license, and DUA. Consequently, the datasets themselves are not openly available without adhering to the specific terms set forth by their respective agreements.

Limitations

Given the descriptive nature of this paper and our stated ambition of enhancing research by lowering barriers to data access, we have no information suggesting that establishing OnetoMap has increased research interest, grants, publications, or abstracts to date. 

The current count and coverage of datasets may be limited, potentially restricting the scope of available research data. Also, maintaining and updating the repository poses challenges, requiring continuous effort and resources to ensure data accuracy and relevance. Additionally, ethical and legal considerations related to data sharing and use must be meticulously managed to prevent misuse. Finally, there is also a need for user training and support to ensure that researchers can effectively utilize the repository, as navigating and integrating complex datasets can be challenging without adequate guidance.

Future directions

We have focused on developing and maintaining a high-quality meta-data repository until now. Moving forward, we plan to implement several strategies to ensure the datasets remain current and valuable for researchers.

One of our primary goals is to enable automatic annual updates of existing datasets, which will ensure the available datasets remain up-to-date and relevant to the research community. Additionally, we plan to explore the possibility of automatic dataset linkage, where different datasets can be linked together when allowed to provide a more comprehensive picture of the research topic.

Another area of focus will be to provide monthly updates of published papers by our group and other groups using the datasets on the OnetoMap meta-data repository. The goal is to keep the research community informed about new developments and insights that emerge from the analysis performed using the available datasets.

In addition, to facilitate communication and collaboration among potential users of the OnetoMap meta-data repository and its datasets, we plan to create a chat space for users or subscribers. This space will allow users to exchange ideas, ask questions, and share insights.

Finally, we plan to develop small code applets for easy data analysis. These applets will be designed to simplify the data analysis process, making it more accessible to researchers who may not have extensive programming experience.

In summary, our future directions involve a commitment to ensuring that our OnetoMap meta-data repository remains current, functional, and accessible to the research community. These efforts will help to facilitate new discoveries and insights, ultimately leading to advancements in our understanding of healthcare outcomes.

## Conclusions

The OnetoMap meta-data repository contains a curated list of clinical research databases designed to facilitate research collaborations between multiple research groups and the Department of Surgery at the USF, as can be seen from the publications generated by OnetoMap since its inception. Each database entry includes detailed descriptions covering the primary purpose, available variables, and examples of high-impact research and applicable methods. OnetoMap aims to develop a centralized inventory that enables users to efficiently locate datasets with the desired data elements, thereby enhancing the scope and efficiency of their analyses. The repository also highlights datasets with potential linkages to other datasets focused on patients, hospitals, environmental factors, or social determinants of health. By fostering collaborations, OnetoMap seeks to dismantle barriers to knowledge dissemination, making research and information more accessible to improve clinical research. Ultimately, the goal is to enable researchers to evaluate not only specific hospital-related questions but also the broader healthcare environment.

Regarding data access, the repository’s design incorporates several features that make it straightforward and efficient. Firstly, the use of a GitHub-based platform ensures a familiar and user-friendly interface for many researchers, allowing easy navigation and data retrieval. Also, the comprehensive documentation and detailed data dictionaries available on GitHub wikis provide clear guidelines and descriptions for each dataset, reducing the learning curve for new users. By organizing datasets with thorough descriptions, applicable methods, and variable categorizations, researchers can easily understand and utilize the available data. Furthermore, the inclusion of search functionalities and categorization of datasets by type, source, and linkage capabilities help users quickly locate relevant data. Finally, the OnetoMap facilitates ethical compliance by maintaining all necessary documentation. These design and feature choices make the OnetoMap repository an accessible and valuable tool for researchers, promoting efficient data use and collaboration across various studies.
